# Association between Iron Deficiency Anemia and Febrile Convulsion in 3- to 60-Month-Old Children: A Systematic Review and Meta-Analysis

**Published:** 2014-11

**Authors:** Narges Habibian, Abbas Alipour, Abbas Rezaianzadeh

**Affiliations:** 1 Center of Medical Education Studies and Development, Mazandaran University of Medical Sciences, Sari, Iran;; 2 Thalassemia Research Center, Department of Community Medicine, School of Medicine, Mazandaran University of Medical Sciences, Sari, Iran;; 3 Department of Epidemiology, School of Health and Nutrition, Shiraz University of Medical Sciences, Shiraz, Iran

**Keywords:** Febrile, Children, Iron deficiency anemia

## Abstract

Controversy exists regarding the association between Iron Deficiency Anemia (IDA), iron status, and Febrile Convulsion (FC) during childhood. In this article, a systematic review and meta-analysis is conducted in order to determine possible association and the degree of association between these statuses and FC. To identify all studies related to IDA and FC, various references such as MEDLINE (PubMed), Embase (OVID), Web of sciences (Thomson Reuters) and Google scholar were searched (up until 15 January 2013). Heterogeneity was assessed using the Q statistic, Tau^2^, and I^2^. Additionally, subgroup analyses were performed. The outcome of primary interest was the overall Odds Ratio (OR) of FC for IDA and standard mean differences (SMD) of ferritin level. In total, 21 articles were considered to assess the association between IDA and FC. Anemia was more prevalent among the FC patients compared with the controls and the overall OR was 1.52 (95% CI=1.03 to 2.25). In addition, the pooled OR for 17 studies performed in the populations with low and moderate prevalence of anemia was 2.04 (95% CI=1.46 to 2.85). Furthermore, 12 studies assessed the association between the ferritin level and FC. The overall SMD was -0.02 with a 95% CI of -0.09 to 0.06. Besides, the pooled SMD of ferritin was -0.57 (95% CI=-0.7 to -0.46) in 6 studies reporting no difference between the FC and the control group with respect to temperature. IDA was associated with a moderate increased risk of FC in children, particularly in the areas with low and moderate prevalence of anemia.

## Introduction

Febrile Convulsions (FC) refer to the convulsions that occur in children between the ages of 6 months and five years, with body temperature of 38ºC or higher not resulting from Central Nervous System (CNS) infection or any metabolic imbalance without any prior afebrile seizures. This condition occurs in 2-5% of the children who are neurologically healthy.^1^ The precise cause of FC is not known, but several genetic and environmental factors have been implicated.^2^ The maximum age of FC occurrence is 14-18 months, which overlaps with the maximum prevalence of Iron Deficiency Anemia (IDA) which is 1-2 years old.^3^

IDA is the most common nutritional deficiency in the world. Iron is an important micronutrient which is used by roughly all the cells in the human body. It is well understood that iron is a cofactor for several enzymes in the body and has a role in the neurotransmitters production and function, hormonal function and DNA duplication.^4^

Iron deficiency stimulates the function of neurons and, consequently, increases the risk of convulsions.^3^^,^^5^ Similar conditions are observed in Attention Deficit Hyperactivity Disorder (ADHD) and Restless Leg Syndrome (RLS).^6^ Animal studies have shown the pathophysiology of this malfunction. The relationship between IDA and FC is unknown. While some studies have shown IDA as a risk factor for the development of FC,^7^^-^^10^ this relationship has not been confirmed by other studies.^5^^,^^11^ On the other hand, few reports have claimed that IDA may have a protective effect on FC development.^2^^,^^12^^,^^13^

With respect to the high prevalence of FC and IDA in children and considering the fact that IDA is a probable risk factor for FC occurrence, as well as controversy in previous studies on this subject, this meta-analysis is carried out to determine the role of IDA in FC development by comparing IDA and ferritin level between FC patients and controls. 

## Methods

*Data Source*

MEDLINE (PubMed), Embase (OVID), Web of sciences (Thomson Reuters) and Google scholar were searched by NH and AA for abstracts using a combination of text words. The following index (MeSH) terms were sought for ‘‘Iron deficiency anemia” [MeSH Terms] OR ‘‘iron status’’ [All Fields] AND ‘‘febrile convulsion’’ [MeSH Terms] OR ‘‘febrile seizure’’ [All Fields]. No limitations or time period restrictions were applied during the search. A manual search of the bibliography of the retrieved papers was also carried out and the experts in this field were contacted for additional references. The latest date for the search was on January 15, 2013. 

*Study Selection*

**Figure 1:**
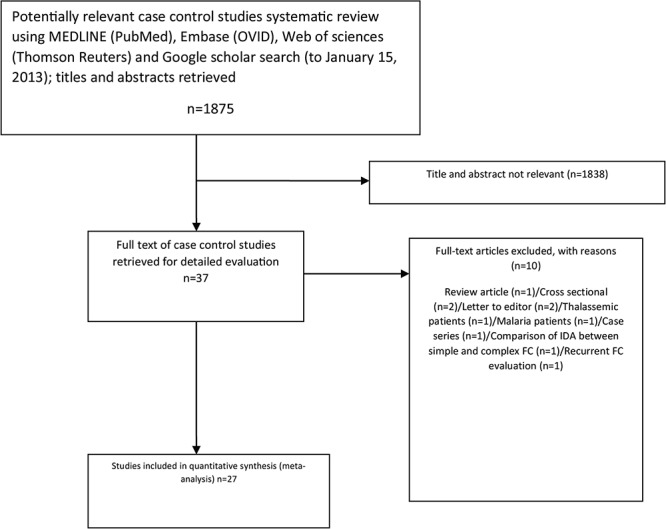
MOOSE flowchart showing selection of studies for meta-analysis of association between Iron Deficiency Anemia and Febrile Convulsion.

The analysis was restricted to human studies and no restriction was placed with respect to the language (figure 1). Studies, which did not follow a case-control or comparative design with clear comparative groups of cases with seizures and controls without seizures were excluded from the analysis. The studies in which the cases did not have a fever, the cases and controls having other hematologic conditions, or the controls which have had any kind of seizure were excluded. Additionally, the studies that proposed no specified criteria for defining iron deficiency were excluded. The abstracts of these articles were thoroughly checked in order to select the most appropriate investigation. Attempts were also made to identify additional articles by searching the reference lists of the studies. Authors were contacted for additional information if data were not reported in a suitable format for data synthesis. Eventually, EndNote software was used to merge the retrieved citations and eliminate the duplications.

*Data Extraction*

Among the selected articles, the data in the study design, the raw number of FC patients and controls who had or had not experienced IDA, the patients’ age and sex, the diagnostic criteria used for definition of IDA, and source of cases and controls were extracted independently. Any discrepancy on the suitability for inclusion of a study was resolved by discussion among the authors. ******

*Statistical Analysis*

In this analysis, STATA (v. 10) and the inverse variance model was used to calculate the overall Odds Ratio (OR), 95% confidence interval (CI) and test statistic for the relationship. Because serum ferritin was continuous measurements, we used standard mean differences (SMD) as the effect size.

Statistical heterogeneity of studies was assessed through the calculation of tau^2^ and I^2^. A random effects model was applied unless I^2^ was <25% in which a fixed effects model would be used.^14^ To dissipate any heterogeneity, subgroup analysis was performed on IDA definition. Generation of a funnel plot and the Egger and Begg P value allowed determination of the potential publication bias. In addition, the “trim-and-fill” method was utilized to obtain the estimates of ORs corrected for a possible publication bias (metatrim command). Furthermore, the quality of the studies was evaluated using the Newcastle-Ottawa Scale (NOS).^15^ To assess the effects of the study characteristics on risk, random-effects meta-regression analyses were performed on the ORs adjusted for the year of study and each study characteristics was assessed by the Newcastle-Ottawa Scale for case-control studies.

## Results

*Included Studies*

The initial search for studies involving the association between FC and anemia (or iron status) yielded 1,875 articles. Yet, after reviewing the abstracts and exclusion of irrelevant and duplicated articles, 37 articles remained in the analysis (figure 1). Out of the 37 examined articles, one review article,^16^ two cross-sectional studies,^17^^,^^18^ two letters to the editor,^4^^,^^19^ two case-control studies which included thalassemic^20^ and malaria patients,^21^ one case series,^22^ one study that compared simple and complex FC regarding IDA,^23^ one study which included FC patients and evaluated the association between the paraclinical findings (e.g. anemia) and seizure recurrence,^24^ were excluded. Additionally, one study assessing the iron status via serum iron^25^ was also excluded. Furthermore, one article^7^ was counted as two studies since it contained two different control groups (hospital and population) and the association was assessed twice. The information about the selected studies can be found in table 1 and the assessed quality of each study using NOS is presented in table 2.

**Table 1:** Information about the included articles in the final meta-analysis

**First author and year of publication**	**Iron deficiency anemia (IDA) criteria**	**Kind of febrile convulsion (FC)**	**Cases**	**Controls**	**OR** **(95% CI)**	**Case source**	**Control source**	**Age range and (mean age of cases)**
Kobrinsky et al.,^12^ 1995	Hb<11 g/dl	Simple FC	26	25	0.39 (0.1-1.5)	Hospital	Hospital	6-36 m/o (18.9±7.6)
Pisacane et al.,^7^ 1996	Hb<10.5 g/dl, MCV<70 fL, Serum Iron<5.4 µmol/L	unknown	146	146	2.57 (1.43-4.59)	Hospital	Hospital	6-24 m/o (15±5.6)
Pisacane et al.,^7^ 1996	Hb<10.5 g/dl, MCV<70 fL, Serum Iron<5.4 µmol/L	unknown	146	147	3.3 (1.78-6.11)	Hospital	Population	6-24 m/o (15±5.6)
Daoud et al.,^26^ 2002	Hb<11 g/dl	First FC Simple (n=66) Complex (n=9)	75	75	1.46 (0.73-2.93)	Hospital	Hospital	18.8 m/o
Guzman et al.,^9^ 2005	Hb<2SD of normal value for age	First FC	40	40	3.89 (1.53-9.87)	Hospital	Hospital	3 m/o–5 y/o
Rehman et al.,^8^ 2005	Hb<10 g/dl	First FC	30	30	7.67 (2.42-24.24)	Hospital	Hospital	8-36 m/o (22.97±9.52)
Al-Zwaini et al.,^27^ 2006	Hct<33%	First and recurrent FC	100	100	2.66 (1.46-4.84)	Hospital	Hospital	6-60 m/o (25.8±15.19)
Talebian et al.,^28^ 2008	Hb<2SD of normal value for age	Simple FC (n=56) Complex FC (n=4)	60	60	0.62 (0.23-1.63)	Hospital	Hospital	under 5 y/o
Abaskhanian et al.,^13^ 2009	Hb and Hct<2 SD of normal values for age^a^	First simple FC	100	100	0.48 (0.27-0.85)	Hospital	Hospital	6 m/o-5 y/o (21.9±14.1)
Hartfield et al.,^3^ 2009	Hb<11 g/dl, MCV<70 fL and RDW>15.6%	Simple or complex FC	361	390	1.42 (0.74-2.73)	Hospital	Hospital	6-36 m/o
Bidabadi et al.,^11^ 2009	Hb and Hct <2 SD of normal values for age^a^	First FC Simple (n=132) Complex (n=68)	200	200	0.85 (0.57-1.26)	Hospital	Hospital	6 m/o-5 y/o (22.86-12.86)
Abdurrahman et al.,^29^ 2009	Hb<10.5 g/dl, MCV<70 fL, serum iron<22 μg/dl, and TIBC>400 μg/dl	First FC	112	120	3.44 (1.73-6.83)	Hospital or who visited private office of the authors	unknown	5 m/o-4 y/o
Amirsalari et al.,^30^ 2010	Hb<10.5 g/dl	First FC	132	88	0.43 (0.12-1.56)	Hospital	Hospital	9 m/o-5 y/o
Sherjil et al.,^31^ 2010	Hb<9 g/dl, serum ferritin<7, MCV<65, MCHC<28	Unknown	157	153	1.92 (1.14-3.23)	Hospital	Hospital	6 m/o-6 y/o
Jun et al.,^32^2010	Hb<10.5 g/dl	First and recurrent FC Simple (n=59) Complex (n=41)	100	100	1.64 (0.91-2.98)	Hospital	Hospital	15.8±6.1 m/o
Kumari et al.,^10^ 2012	Hb<11 g/dL, serum ferritin<12 and RDW>15%	Simple FC	154	154	5.34 (3.27-8.74)	Hospital	Hospital	6 m/o-3 y/o (17.5±8.81)
Derakhshanfar et al.,^2^ 2012	Hb and Hct<2 SD of normal values for age^a^	Unknown	500	500	0.57 (0.45-0.74)	Hospital	Hospital	6 m/o-5 y/o (26.49+12.65)
Heydarian et al.,^5^ 2012	Hb<10.5 g/dl	First simple FC	120	120	1.04 (0.61-1.75)	Hospital	Hospital	6 m/o-5 y/o (20.7±14.8)
Zareifar et al.,^35^ 2012	Hb<2 SD of normal value for age	Simple FC	300	200	0.42 (0.29-0.6)	Hospital	Hospital	6 m/o-5 y/o (26.4±13.8)
Majumdar et al.,^33^ 2012	Hb<11 g/dl, MCV<70 fL, MCH<27 pg, ferritin<12 μg/dl, serum ferrous<60 μg/dl, TIBC>450 μg/dl, transferrin<250 mg/dl	First FC	50	50	6.29 (2.52-15.7)	Hospital	Hospital	6 m/o-6 y/o
Sadeghzadeh et al.,^34^ 2013	Hb<10.5 g/dl	First and recurrent FC Simple (n=70) Complex (n=30)	100	100	1.3 (0.64-2.64)	Hospital	Health care center	6 m/o-3 y/o
Mahyar et al.,^36^ 2006	-	First simple FC	20	20	-	Hospital	Hospital	9-24 m/o
Salehi et al.,^37^ 2009	-	First FC	90	90	-	Hospital	Hospital	9m/o-5 y/o (1.6±1.2)
Momen et al.,^39^ 2010	Hb<11 g/L, MCV<72 fL, ferritin<20 μg/dL and TIBC<440 μg/dL	First simple FC	50	50	-	Hospital	Hospital	9 m/o-5 y/o
Vaswani et al.,^40^ 2010	hemoglobin<11 g /dL, MCV<70 fL, MCH<27 pg and serum ferritin<12 μg/dL	First FC	50	50	-	Hospital	Hospital	6 m/o-6 y/o (1.73±0.94)
Talebian et al.,^38^ 2011	-	Unknown	40	40	-	Hospital	Hospital	6 m/o-5 y/o (24.8±13.95)

OR (95% CI): Odds ratio with 95% confidence intervals; SD: Standard deviation; Hb: Hemoglobin; Hct: Hematocrit; MCV: Mean corpuscular volume; MCHC: Mean corpuscular hemoglobin concentration; TIBC: Total iron binding capacity; m/o: Months old; y/o: years old; ^a^Hb<10.5 g/dl for 6–24 months and <11.5 g/dl for 24–60 months old, Hct<33% for 6–24 months and <34% for 24–60 months old, MCV<70 fL for 6–24 months and <75 fL for 24–60 months old, MCH<23 pg for 6–24 months and <24 pg for 24–60 months old, MCHC<30 g/dl for 6–24 months and <31 g/dl for 24–60 months old, RBC<3.7×10^6^ for 6–24 months and <3.9×10^6^ for 24–60 months old, serum iron concentration <40 mg/dl before 1 year old and <50 mg/dl after 1 year old, PF<7 mg/l, and TIBC>430 mcg/dl

**Table 2:** Quality assessment of the included studies

	** Quality Indicators From Newcastle-Ottawa Scale^a^**								
	**1**	**2**	**3**	**4**	**5A**	**5B**	**6**	**7**	**8**
Kobrinsky et al.,^12^ 1995	Yes	No	No	Yes	Yes	No	No	Yes	No
Pisacane1 et al.,^7^ 1996	Yes	No	No	Yes	Yes	No	Yes	Yes	No
Pisacane2 et al.,^7^ 1996	Yes	No	Yes	Yes	Yes	No	Yes	Yes	No
Daoud et al^26^, 2002	Yes	No	No	Yes	Yes	No	Yes	Yes	No
Guzman et al.,^9^ 2005	Yes	No	No	Yes	Yes	No	No	Yes	No
Rehman et al.,^8^ 2005	Yes	No	No	Yes	Yes	No	No	Yes	No
Al-Zwaini et al.,^27^ 2006	Yes	No	No	Yes	Yes	No	No	Yes	No
Talebian et al.,^28^ 2008	Yes	No	No	Yes	Yes	No	No	Yes	No
Abaskhanian et al.,^13^ 2009	Yes	No	No	Yes	Yes	No	No	Yes	No
Hartfield et al.,^3^ 2009	Yes	No	No	Yes	Yes	No	No	Yes	No
Bidabadi et al.,^11^ 2009	Yes	No	No	Yes	Yes	yes	No	Yes	No
Abdurrahman et al.,^29^ 2009	Yes	No	No	Yes	Yes	Yes	No	Yes	No
Amirsalari et al.,^30^ 2010	Yes	yes	No	Yes	Yes	No	No	Yes	No
Sherjil et al.,^31^ 2010	Yes	No	No	Yes	Yes	No	No	Yes	No
Jun et al.,^32^2010	Yes	No	No	Yes	Yes	No	No	Yes	No
Kumari et al.,^10^ 2012	Yes	No	No	Yes	Yes	No	No	Yes	No
Derakhshanfar et al.,^2^ 2012	Yes	No	No	Yes	Yes	No	No	Yes	No
Heydarian et al.,^5^ 2012	Yes	No	No	Yes	Yes	No	No	Yes	No
Zareifar et al.,^35^ 2012	Yes	No	No	Yes	Yes	Yes	No	Yes	No
Majumdar et al.,^33^ 2012	Yes	No	No	Yes	Yes	No	No	Yes	No
Sadeghzadeh et al.,^34^ 2013	Yes	No	Yes	Yes	Yes	No	No	Yes	No

^a^1: Indicates cases independently validated; 2: Cases are representative of population; 3: Community controls; 4: Controls have no history of Febrile Convulsion; 5A: Study controls for age; 5B: Study controls for additional factor(s) e.g. Iron supplement, Temperature, etc.; 6: Ascertainment of exposure by blinded interview or record; 7: Same method of ascertainment used for cases and controls; 8: Non-response rate the same for cases and controls

*Anemia in FC Patients vs. Controls*

**Figure 2:**
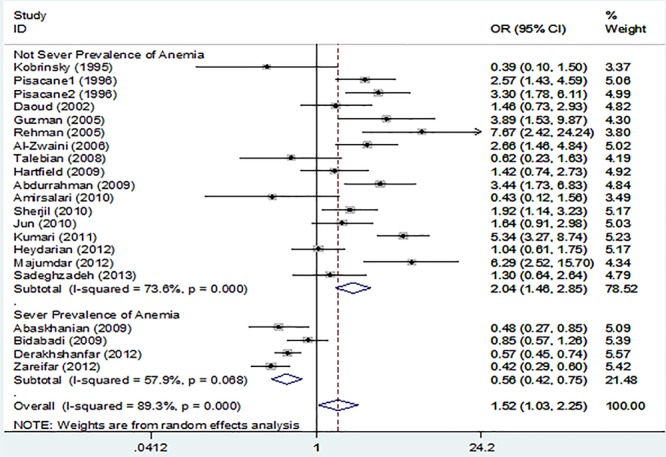
The figure demonstrates the pooled odds ratios and 95% confidence intervals for Iron deficiency anemia when comparing febrile convulsions (FC) patients with control groups and overall and subgroup analysis according to prevalence of anemia in population of studies (higher vs. lower than 40%). The studies are listed based on date

According to the results, a statistically significant association was found between anemia and FC. The overall OR upon the inclusion of all the 21 studies^2,3,5,7-13,26-35^ was 1.52 (95% CI=1.01 to 2.28) [I^2^=89.7; P<0.001; tau^2^=0.72] (figure 2).

The evidence of publication bias was provided by a funnel plot. The Egger test was significant for publication bias (P=0.02) but not the Begg test (P=0.43).

The stratified analyses for anemia criteria according to the IDA definition yielded pooled OR estimates of 1.84 (95% CI=1.02 to 3.33; z=2.04; P=0.04) [I^2^=92.7; P<0.001; tau^2^=0.81] for 10 studies with anemia criteria according to iron status and 1.26 (95% CI=0.73 to 2.1; z=0.84; P=0.4) [I^2^=84.6; P<0.001; tau^2^=0.63] for 11 studies with anemia criteria according to Hb and hematocrit (Hct). Furthermore, the pooled OR was 0.56 (95% CI=0.42 to 0.75; z=3.87; P<0.0001) [I^2^=57.9; P<0.001; tau^2^=0.05] for 4 studies performed in the populations with a high prevalence of anemia (>40% in the control groups), but 2.04 (95% CI=1.46 to 2.85; z=4.16; P<0.0001) [I^2^=73.6; P<0.001; tau^2^=0.34] for 17 studies conducted among those with low and moderate prevalence of anemia (<40% in the control groups) (figure 2).

The influence analyses were completed by recalculating the pooled ORs for the sample on multiple occasions, while removing one study at each iteration. These analyses were particularly important because several studies included samples that were substantially larger than most of the other studies and thus could have exerted large effects on the overall effect estimates. For all studies, these analyses yielded ORs ranging from 1.43 (95% CI=0.97 to 2.1) to 1.64 (95% CI=1.1 to 2.4). 

The present analysis also assessed the relationship between the quality of the study, year of the study, IDA definition, FC definition, the prevalence of anemia in the study population and the effect size (log OR) via random effects meta-regression. This analysis showed that even after correction for these elements, the relation between IDA definition (coefficient=0.76; SE=0.36; P=0.04) and the prevalence of anemia in the study population (coefficient=-1.74; SE=0.46; P=0.003) and FC was significant (I^2^=72.7; tau^2^=0.33). Nevertheless, the regression model revealed that none of the NOS scores and the year of the study was significantly related to OR.

*Ferritin Level in FC Patients vs. Controls*

**Figure 3:**
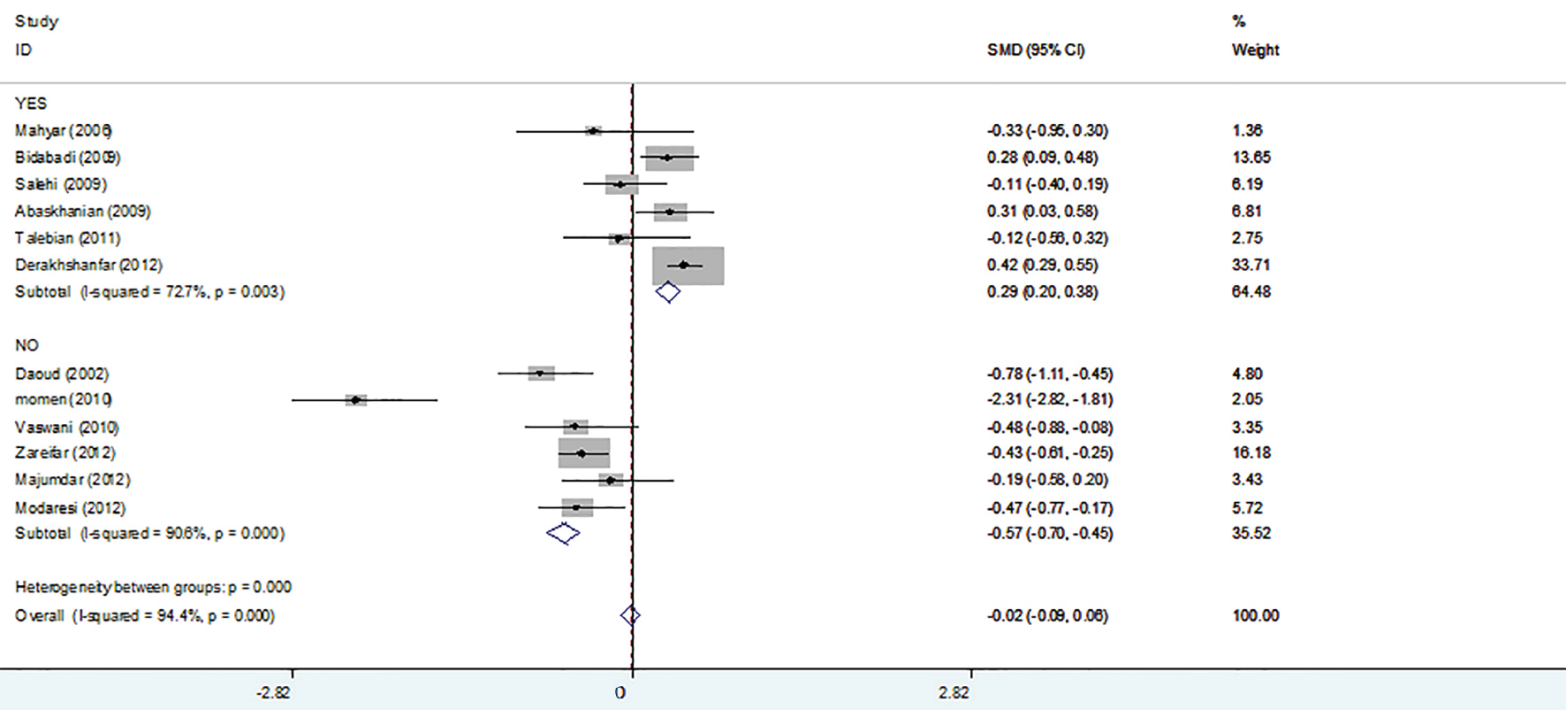
The figure demonstrates standard mean difference (SMD) of ferritin level and 95% confidence intervals when comparing febrile convulsions (FC) patients with control groups and overall and subgroup analysis according to significance difference between temperature of case and control. The studies are listed based on date.

Data on ferritin level were available for 12 studies.^2,11,13,26,33,35-41^ Four studies (Daoud et al.,^26^ Momen et al.,^39^ Vaswani et al.,^40^ and Modaresi et al.^41^) reported a significant decrease in the ferritin level in FC patients. On the other hand, three studies (Abaskhanian et al.,^13^ Bidabadi et al.,^11^ Derakhshanfar et al.^2^) reported a significant increase in the ferritin level in the FC patients compared with controls. The results of a random effects model for the 12 case-control studies included in the present meta-analysis are presented in figure 3. The overall Standard Mean Difference (SMD) with a 95% CI was -0.02 (-0.09 to 0.06; z=0.47; P=0.64) [I^2^=94.4; P<0.001; tau^2^=0.31].

The publication bias was evaluated by a funnel plot. Neither Egger test (P=0.54) nor Begg test (P=0.49) was statistically significant for the publication bias.

The pooled SMD of ferritin was -0.57 (with 95% CI of -0.7 to -0.46; z=9.23; P<0.001) [I^2^=90.6; P<0.001; tau^2^=0.26] for the six studies reporting no difference between FC and control groups regarding the temperature. On the other hand, it was measured as 0.29 (95% CI=0.2 to 0.38; *z*=6.26; P<0.001) [I^2^=86.6; P<0.001; tau^2^=0.04] for the other 6 studies showing a significant difference between FC and control groups regarding temperature (figure 3).

## Discussion

The results revealed a statistically significant relationship between FC and IDA; IDA was 1.52 times more prevalent among the FC patients compared with controls. We observed a significant heterogeneity in this meta-analysis. Some parts of this heterogeneity can be explained by variations in the demographic characteristics, the primary illness resulting in fever, fever severity (temperature), family history of seizures, selection bias (especially in the control groups), etc. (table 1).

Yet, meta-regression and subgroup analyses showed that the state of IDA prevalence in the control group, which should reflect the prevalence of IDA in the general population, could be one of the main reasons for the observed heterogeneity. Of course, this relationship was more significant and heterogeneity showed a decreasing trend in the subgroup analysis which did not have a high prevalence of IDA (<40%) (OR: 2.07). In other words, the association between IDA and FC was significant in the regions with a low/medium prevalence of IDA, but not in those with a high prevalence of IDA (>40%).

One possible explanation is that when the rate of IDA is high in a particular population, the difference between the ratio of IDA in FC patients and controls is not high enough to show a significant difference. Moreover, most studies with high rates of IDA were done in Iran. This indicates the role of genetics, besides IDA, as a cofactor in FC.^2^ In other words, since FC is a multifactorial disease, it can be concluded that genetics trigger the effects of IDA on FC.^42^^,^^43^ Furthermore, reports have suggested that FC development is associated with the socio-economic level of patient’s family.^4^^,^^44^ It is obvious that the socio-economic status is associated with IDA; therefore, it is anticipated that IDA has an intermediate effect on FC and is a negative confounder with other factors related to low levels of socio-economic status.^45^ Overall, the associated factors with low socio-economic status includes zinc,^46^^,^^47^ magnesium.^48^^,^^49^ selenium,^48^^,^^50^ copper deficiencies^47^ and high levels of lead.^8^

The ferritin level is affected by the severity of fever and increases with fever. In some studies, the severity of fever was not similar in the FC patients and controls. Consequently, ferritin level could not be compared between the two groups. Nonetheless, when groups with similar fever severity (temperature) were compared, ferritin level was found to be lower in the FC children compared with the healthy ones. This is consistent with other findings from our meta-analysis. 

According to the findings, iron deficiency leads to dysfunction of myelination as well as tyrosine and tryptophan hydroxylase synthesis, which are necessary for neurotransmitter production as well as the release of neurotransmitters from vesicles.^21^^,^^51^ The role of iron has also been documented in the production of serotonin, dopamine, and Gamma-Butyric Acid (GABA).^5^

Bradford Hill’s criterion is among the best for showing a causative relationship.^52^^,^^53^ Generally, it is difficult to draw conclusions about the causal relationships in observational studies. However, this meta-analysis could address some of the Hill’s criteria.^53^ The strength of the association was 2.07 (in populations with low and moderate prevalence of anemia) and the criteria could be accepted. Some studies have revealed the temporality and earlier occurrence of IDA compared to FC. In most studies, the primary event was the first FC and, as a result, we can accept that IDA/iron deficiency occurred before FC. Yet, it is difficult to draw such conclusion in the studies where the evaluated event was both the first and the recurrent FC. The association between IDA/iron deficiency and FC is consistent with the current knowledge and theories (coherence criteria). The biological plausibility evidence demonstrates the role of iron as an important cofactor for normal functioning of the brain neurotransmitters. However, limited data are available on the biological gradient between the cause (IDA) and the effect (FC). Therefore, further study on this topic is recommended. Since FC is a multi-factorial disease, specificity criteria cannot be adhered. According to these criteria, IDA should only cause an effect (FC) to be concluded as a cause for FC. Evidence regarding the administration of iron for FC children has also been controversial and requires more research.^20^^,^^54^^,^^55^ Overall, it can be stated that IDA causes FC through a similar mechanism that causes ADHD, Breath Holding Spells (BHS) and RLS (analogy).^6^

In addition to the observational nature of the articles used here, this meta-analysis had other limitations. First, the quality of some of the articles was not high enough. For example, the characteristics of the drop out patients were not clear. Besides, in most of the studies, data gathering was not done in a blinded fashion and selection of the patients was hospital-based rather than population-based.^56^ Additionally, the patients were not selected randomly in few studies. Furthermore, in a few studies, the two study groups were not similar regarding the important factors and the role of the confounding variables was not well controlled. Thus, we attempted to control the role of the quality of these items using meta-regression. Another limitation of this meta-analysis was different markers and definitions for IDA and FC. The odds ratio of heterogeneity was large between the studies and some part of this heterogeneity might be related to different definitions of IDA and/or FC. We tried to control this issue by performing a meta-regression analysis and then subgroup analysis. The funnel plot indicated the lack of symmetry and the possibility of bias. However, using “trim and fill” sensitivity analysis, no considerable change was observed in the results indicating the low level of bias in the performed meta-analysis.

Further high-quality studies (cohort or case-control) are recommended on this topic. In future studies, IDA and FC should be defined more precisely and the role of the important factors, such as socio-economic status and the serum levels of zinc, manganese, lead, etc., should be controlled to determine whether the association between IDA and FC is causal or not. After documenting a causative relationship, interventional studies can also be performed to assess the effect of iron administration on the FC patients. 

## Conclusion

Thus far, the combined results of the case control studies suggest that iron deficiency anemia is associated with a moderate increased risk of FC in children, particularly in areas with low and moderate prevalence of anemia. Future work should be carried out on interventional studies and the implication for public health. 
